# Contralateral inclinatory approach for decompression of the lateral recess and same-level foraminal lesions using unilateral biportal endoscopy: A technical report

**DOI:** 10.3389/fsurg.2022.959390

**Published:** 2022-10-31

**Authors:** Dasheng Tian, Bin Zhu, Jianjun Liu, Lei Chen, Yisong Sun, Huazhang Zhong, Juehua Jing

**Affiliations:** Department of Orthopaedics, The Second Affiliated Hospital of Anhui Medical University, Hefei, China

**Keywords:** lumbar, UBE, foraminal and lateral recess stenosis, inclinatory, contralateral

## Abstract

**Objective:**

Unilateral biportal endoscopic (UBE)surgery is being increasingly adopted as a minimally invasive technique. The purpose of the current study was to introduce a novel surgical technique for lateral recess and same-level foraminal decompression by the contralateral inclinatory approach with unilateral biportal endoscopy(CIA-UBE) at the lumbar level.

**Methods:**

Between January 2020 and February 2022, 10 patients suffering from lateral recess and same-level foraminal stenosis at the lumbar level underwent UBE surgery by contralateral inclinatory approach (CIA-UBE). Magnetic resonance imaging (MRI) scans were examined after surgery to measure the cross-sectional area (CSA) of the spinal canal (CSA-SC), the CSA of the intervertebral foramen (CSA-IVF), and the CSA of the facet joint (CSA-FJ). Postoperative radiologic images using computed tomography (CT) were obtained to investigate the existence of facet joint violation. Clinical outcomes were assessed using Oswestry Disability Index (ODI) scores and visual analogue scale (VAS) scores for buttock and radicular pain.

**Results:**

Ten levels were decompressed, and the mean age of the patients was 56.92 ± 13.26 years. The mean follow-up period was 7.60 ± 4.47 months. The average operative time was 85.14 ± 25.65 min. Postoperative CT and MRI revealed ideal neural decompression of the treated segments in all patients. CSA-IVF and CSA-FJ improved significantly, indicating good foraminal and lateral recess decompression with less damage to facet joints. Preoperative VAS and ODI scores improved significantly after surgery.

**Conclusion:**

CIA-UBE may be an effective surgical treatment of the lateral recess and same-level foraminal stenosis at the lumbar level, which provides successful surgical decompression for traversing and exiting nerve roots with a better operative view and easier surgical manipulation. This approach may also help to maximize the preservation of the facet joint.

## Introduction

Lumbar lateral recess and same-level foraminal stenosis is a common disease in which degenerative changes of the vertebral column cause entrapment of traversing and exiting nerve roots (1). There are currently two major surgical treatment options for this disease: decompression with spinal fusion and decompression without fusion (2, 3). However, several disadvantages of fusion surgery, such as junctional problems, instrumental failures, pseudoarthrosis, and chronic back pain due to iatrogenic trauma, have been reported (4–6). Thus, researchers have introduced decompression without fusion using endoscopic spinal surgery (7). However, for the lateral recess and same-level foraminal stenosis, the disadvantage is that proper decompression is difficult without destroying the facet joint due to the two-level nerve roots (one nerve root at the lateral recess and another nerve root at the same level foraminal region) by endoscopic surgery.

Recently, several authors have introduced UBE surgery as a minimally invasive therapeutic option (8–10). Although UBE surgery has been developed with a wider view and more degrees of freedom, significant facet joint violations may develop after ipsilateral laminectomy, especially in areas around the lateral recess and foraminal region (11). A contralateral sublaminar approach has already been introduced in UBE surgery to preserve facet joints during decompression (12, 13). However, the current literature does no describe the contralateral inclinatory approach with unilateral biportal endoscopy at the lumbar level.

We attempted a contralateral inclinatory approach by applying a UBE surgery system to treat lumbar lateral recess and same-level foraminal stenosis pathologies. The purpose of the present study was to introduce the surgical technique of CIA-UBE and present preliminary radiologic and clinical results. To the best of our knowledge, this is the first report to describe the lumbar CIA-UBE technique at the lumbar level with patients in prone positions.

## Materials and methods

Between January 2020 and February 2022, a single surgeon team performed 864 UBE surgical procedures for lumbar degenerative diseases. Among the total 864 patients, 10 patients treated *via* CIA-UBE for lumbar lateral recess and same-level foraminal stenosis were included in this study. Demographic characteristics, classification of pathologies, distribution of operation level, operative time, and surgical complications were reviewed.

**Figure 1:**
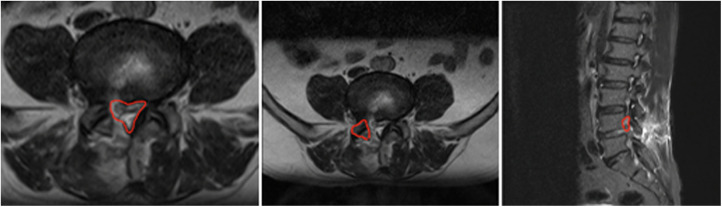
Method of the measurement of CSA-SC, CSA-IVF, and CSA-FJ.

The clinical results were evaluated and compared preoperatively and postoperatively using Oswestry Disability Index (ODI) and the visual analogue scale (VAS) scores for buttock and radicular pain. Pre- and postoperative radiologic images (computed tomography [CT] and magnetic resonance imaging [MRI]) were taken and compared. Preoperative CT and MRI images were examined for the extent of lateral recess and same-level foraminal compression. Postoperative CT and MRI images were recorded to evaluate the adequacy of decompression on the third day after surgery. For the morphometric analysis, the cross-sectional area (CSA) of the spinal canal (CSA-SC), the CSA of the intervertebral foramen (CSA-IVF), and the CSA of the facet joint (CSA-FJ) at the level of foraminal decompression were measured with T2-weighted MRI. CSA-SC was measured using an imaginary line encircling the area between the facet and the lamina. CSA-IVF was measured using an imaginary line around the neural foramen on the symptomatic side of the parasagittal cuts. CSA-FJ was measured using an imaginary line surrounding the facet joint at the affected foraminal compression. All areas were expressed in square millimeters (Figure 1).

## Statistical analyses

Statistical calculations, including means and standard deviations, were obtained using SPSS version 17.0. Paired *t*-tests were used to compare the differences in each parameter of the perioperative outcome. Statistical significance was established at a *p*-value of less than 0.05.

### Indications and contraindications

CIA-UBE was indicated in the patients suffering from unilateral radiculopathy with a diagnosis of degenerative lumbar spinal stenosis at two contiguous levels (one nerve root at the lateral cress and another nerve root at the adjacent level in its foraminal region), which correlated to the neurologic distribution of pain and dysesthesia. All enrolled patients have suffered from unilateral radiculopathy with associated neurogenic claudication and have undergone all conservative measures including bed rest, physiotherapies, and medications, for a minimum of 6 weeks with no alleviation of symptoms. All patients underwent selective nerve root block, indicating that both lesions were pathologic.

The exclusion criteria were the presence of segmental instability, spinous process deviation or hypertrophy, severe kyphosis or rotatory scoliosis, central stenosis with bilateral leg pain, and patients with extraforaminal ruptured discs.

### Preoperative evaluation

Patients were routinely evaluated with anteroposterior, lateral, oblique, and dynamic x-rays to assess spine alignment, disc space height, foraminal bony encroachment, and instability. Additional radiographic evaluations, such as MRI and CT, were performed to evaluate the degree of foraminal stenosis and acquire detailed information about the facet joint, such as the degree of joint hypertrophy, tropism, size and shape of the bony spur, and inclination angle of the spinous process. This allowed the surgeon to determine the amount of facet joint resection and approach angle for ideal decompression with the preservation of segmental stability.

## Surgical technique

### Instruments used in CIA-UBE

During the operation, we used a 30° 4-mm-diameter arthroscope (Smith & Nephew, USA), a 90° 3.75-mm radiofrequency ablator, and a 1.4-mm microablator radiofrequency probe (Bonss Medical, Jiangsu Bonss Medical Technology Company., Ltd., China). We also used instruments such as 3-mm-diameter straight and curved round burr (Guizhou Zirui Technology Co. Ltd., China), 3-mm curved curettes, and 3-mm straight and curved chisels.

### Surgical procedure

#### Anesthesia and patient positioning

The patient was placed in a prone position with flexion on a radiolucent frame under general anesthesia. The abdomen was relaxed using an H-shaped pillow to avoid increased abdominal pressure. The entire posterior back was prepared with an antiseptic solution and draped with a waterproof surgical drape.

#### Skin incisions and making portals

**Figure 2:**
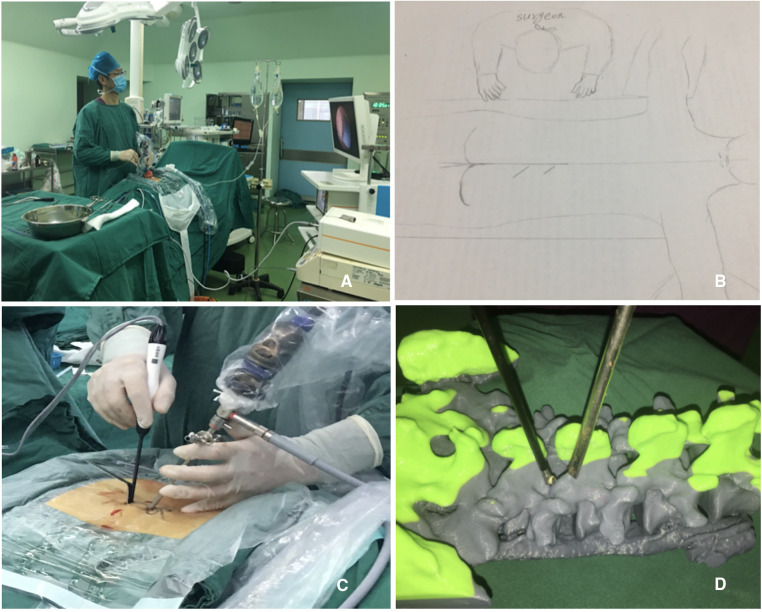
Surgeon’s operative position and schematic illustration of operative setup. (**A**) If the patient has a right side lesion, the operating surgeon stands on the left side and the procedure is performed on the right (lesion) side *via* inclinatory operative trajectory (position for a right-handed surgeon); (**B**) schematic illustration of the operation setup; (**C**) intraoperative views of contralateral Inclinatory approach to the lesion side over the midline of spinous process and the angle of scope and instruments; and (**D**) inclinatory operative trajectory is simulated on the artificial lumbar spine model.

**Figure 3:**
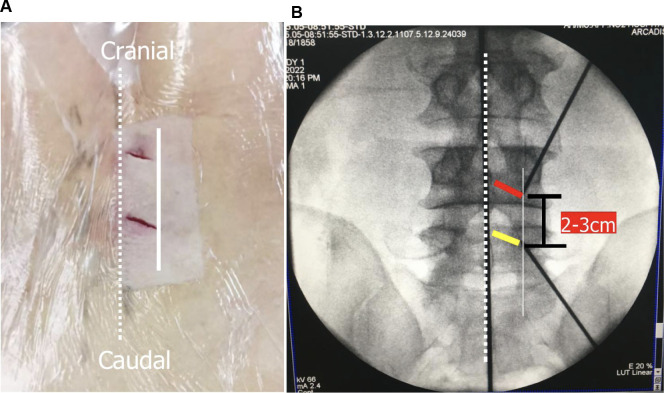
Initial placement of two portals and related anatomy. (**A**). Skin entry points for two portals; and (**B**) position of two portals on an anteroposterior view of x-ray (yellow line: the site for placement of the cranial portal; red line: the site for placement of the caudal portal; dotted line: midline; white line: medial pedicular line).

The contralateral side means the surgeon should stand on the opposite side of the lesion, and two portals were created at the lesion side over the spinal process. If the patient had a right side lesion, the operating surgeon stood on the left side, and the procedure was performed on the right (lesion) side *via* an inclinatory operative trajectory (Figure 2). Under the guidance of C-arm fluoroscopy, two skin incisions were made in the vicinity of the spinous process. The first 0.5 cm-long skin incision for a cranial portal (viewing portal) was made at the level of the lower third of the upper lamina, while the other 1 cm-long skin incision for a caudal portal (working portal) was made at the level of the upper third of the pedicle of the distal vertebra on the C-arm lateral view. Both incisions were made obliquely along the multifidus muscle, and the distance between these two incisions was about 2–3 cm (Figure 3).

#### Insertion of the endoscope and preparation of the surgical field

**Figure 4:**
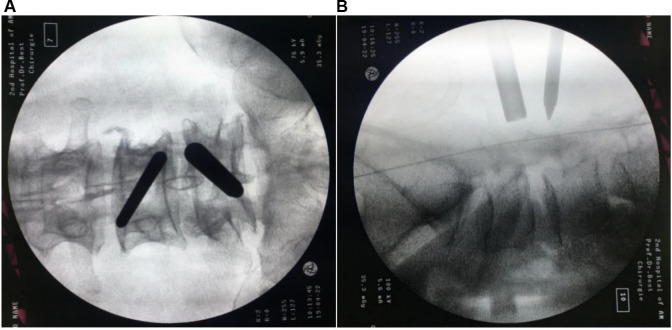
Intraoperative fluoroscopic confirmation of metal rods. Triangulation of metal rods is done at the docking point (the medial of facet joint) under the C-arm: anteroposterior (**A**); and lateral views (**B**).

Serial dilators were passed down along the spinous process and the lamina to dissect the back muscle and acquire operative space. After triangulation with the instruments on the margin of the superior laminar and medial points of the facet joint, the localization was confirmed with anteroposterior and lateral views (Figure 4). A 30° endoscope was inserted through the viewing portal, and a 1.7 m-high saline irrigation system from the operating room floor was applied to create the initial working space. Surgical instruments were inserted through the caudal working portal after inserting the cannula.

#### Laminotomy for making interlaminar working window

Soft tissues overlying the lamina and the ligamentum flavum were ablated to expose the bone edge in the targeted interlaminar space. After complete exposure of the medial point of the facet joint, the inferolateral portion of the upper lamina, and the superolateral part of the lower lamina, keyhole laminotomy was performed with endoscopic drills and Kerrion punches. The medial boundary of the working zone was the spinolaminar junction of the adjacent lamina. Because the proximal origin of the ligamentum flavum is Y-shaped, laminoplasty of the upper laminae should be extended more cranially on the lateral border until the flavum edge is freed. The laminotomy of the lower was performed until full exposure of the ligamentum flavum. The operator should try to make the keyhole wide enough for easier handling of endoscopic instruments, and the deeper ligament flavum should be preserved to protect the neural structure during drilling.

**Figure 5:**
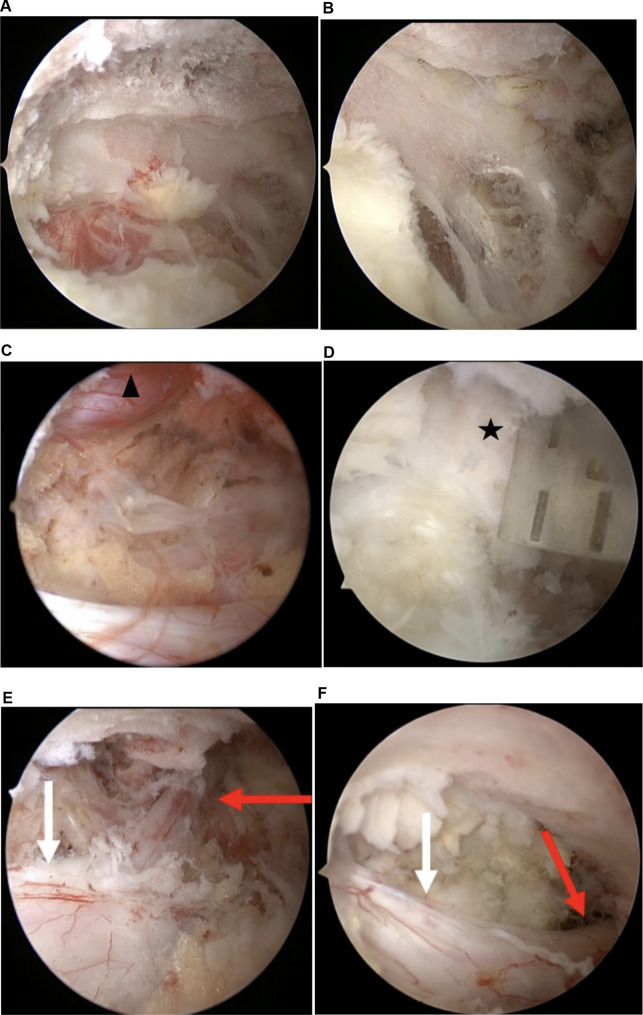
Operative illustrations in the endoscopic view. (**A**) Laminotomy of the crania; (**B**) laminotomy of the cauda; (**C**) cranial tip of the superior articular process is cut by an angled chisel; (**D**) decompressed exiting root in foraminal is observed; (**E**) thecal sac and shoulder margin of traversing root were revealed; (**F**) decompressed traversing root is demonstrated (black Asterisk: cranial tip of the superior articular process; black triangle: L5 exiting root; red arrow: L5 traversing root; black arrow: thecal sac).

When the laminotomy of the upper and lower laminae was finished, by manipulating and tilting the endoscope, the undercutting of the medial point of the facet joint could be achieved by using a bendable 3-mm diamond burr. Thereafter, the interarticular plane of the superior articular process was revealed after the remnant thin bony eggshell was removed by a curette. After determining the medial part of SAP and the lateral recess, a thin bone osteotome, an up-curved chisel, and a Kerrison laminectomy punch can be used to cut the osteophytes and unroof the lateral recess (Figures 5A,B).

#### Flavectomy and decompression

After sufficient bony decompression and the plane between the flavum and dura was defined carefully, flavectomy by piecemeal started from the midline of the thecal sac toward the lateral and from the cranial to the caudal. The edge of the flavum ligamentum was dissected from the bone margin with a small Kerrison laminectomy punch and up-curved curettes.

**Figure 6:**
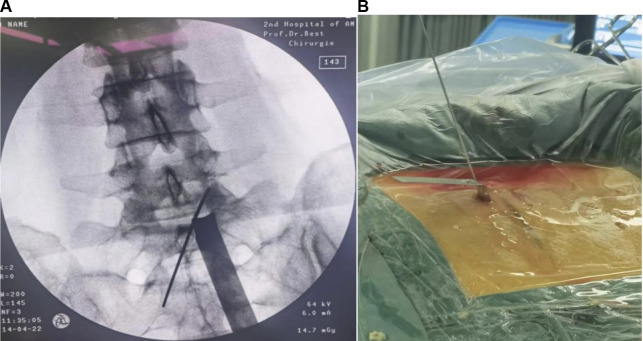
Sufficient decompression in the foraminal area is verified by passing a Kirschner wire probe through the foraminal canal.

After the flavum ligamentum was removed, the spinal canal, along with the lateral margin of the dural sac, was clearly seen. After the nerve root adjacent to the dural sac was identified, an attempt at further facet undercutting down to the medial wall of the pedicle was made to achieve lateral recess decompression until the traversing root was satisfied exposed (Figures 5C–F). In cases where the exiting root was compressed by a protruded or ruptured disc, a discectomy was required. It could be performed using pituitary forceps after adequately exposing the shoulder regions of the traversing root. Cranial foraminal decompression and adhesiolysis proceeded until the exiting root was exposed. In the case of severe foraminal stenosis, which requires wider decompression of the exiting root, the cranial tip of the superior articular process was removed by using a small up-curved chisel. After decompression, sufficient foraminal decompression was verified by passing a ball tip probe through the foraminal canal without any resistance (Figure 6).

#### Wound closure

After meticulous hemostasis was done by radiofrequency coagulation, free traversing and exiting nerve roots were confirmed by gentle retraction with nerve hooks. A drainage catheter was inserted through the working port to prevent postoperative hematoma. Then, the drainage catheter was secured in its place with a suture, and the wounds were closed using two single stitches.

## Results

A total of 10 patients (three men and seven women; mean age 56.92 ± 13.26 years) were enrolled in this study. All patients had only two-level compression (one lateral recess compression and one adjacent foraminal). A total of 10 levels were operated using the aforementioned CIA-UBE in 10 patients. Of these, five patients underwent decompression at L4–L5, and five patients underwent decompression at L5–S1. There were six levels of lumbar disc herniation and four levels of pure foraminal and lateral recess stenosis. No cases were converted to open surgery in any of the patients. None of the patients had dural tears or other adverse events during surgery. The mean operation time was 85.14 ± 25.65 min, and the mean hospital stay was 4.84 ± 1.26 days. The mean follow-up period was 7.60 ± 4.47 months (Table 1).

**Table 1:** Patients’ demographics and disease characteristics (*n* = 10).

Characteristic	Value
Sex, male:female	3:7
Age (year)	56.92 ± 13.26
Level	
L4–5	5
L5–S1	5
Side (lesions)	
Right	8
Left	2
Disc herniation	
Up-migrated	4
Intervertebral	2
None	4
Operative time (min)	85.14 ± 25.65
Hospital stay (day)	4.84 ± 1.26
Final follow-up period (month)	7.60 ± 4.47
MacNab	
Good	2
Excellent	8

Preoperative VAS and ODI scores improved significantly after the surgeries: VAS scores changed from 8.36 ± 0.65 preoperatively to 0.69 ± 0.45 at the last follow-up visit, while ODI scores changed from 79.56 ± 23.56 to 10.74 ± 5.67 (*p* < 0.05). There were no significant complications after the surgery, such as motor weakness or postoperative hematoma.

**Figure 7:**
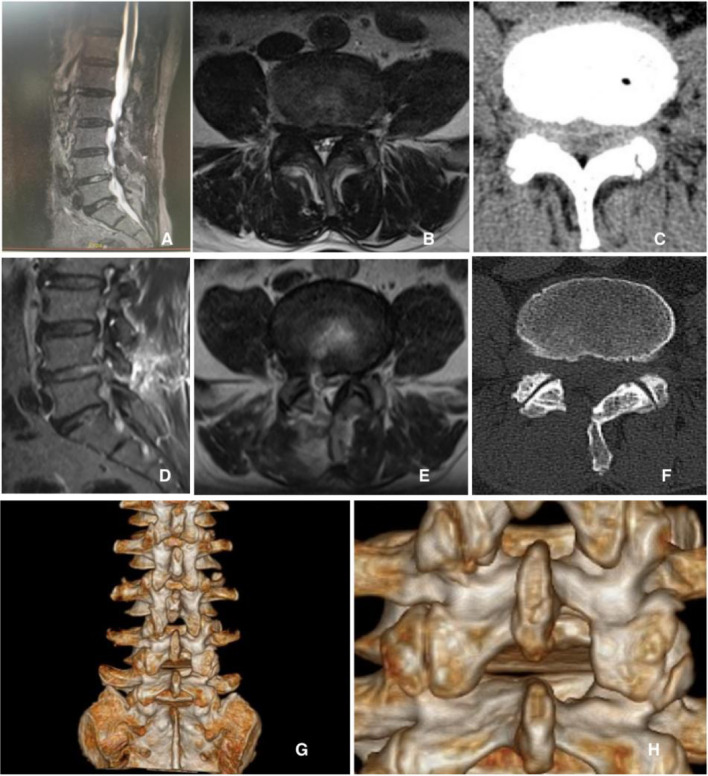
Pre-and postoperative radiologic images of the sixth case. A 58-year-old man presented with lateral recess and cranial level foraminal stenosis on the L4–L5 level. He underwent UBE-CIA on the right side of the L4–L5 level. Preoperative images showed foraminal stenosis on the right side of the L4–L5 level (**A–C**); ideal foraminal decompression with an obliquely undercut facet joint was shown in postoperative images (**D–F**); and three-dimensional computed tomography scan identified the remained facet joints and the range and adequacy of foraminotomy (**G**, **H**).

Postoperative MRI images and CT scans successfully depicted neural root decompression in the lateral recess and foraminal regions of the treated segments in all patients (Figure 7). The mean preoperative and postoperative CSA-CS values were 100.70 ± 32.12 mm^2^ and 143.23 ± 35.12 mm^2^, respectively. The mean preoperative and postoperative CSA-IVF values were 52.35 ± 14.23 mm^2^ and 84.87 ± 19.34 mm^2^, respectively. The mean preoperative and postoperative CSA-FJ values were 216.04 ± 28.23 mm^2^ and 196.64 ± 21.34 mm^2^, respectively (Table 2).

**Table 2:** Morphometric of MRI and clinical outcomes.

	Preoperative	Postoperative (last follow-up visit)	*P*
Cross-sectional area of the spinal canal (CSA-SC) (mm^2^)	100.70 ± 32.12	143.23 ± 35.12	<0.05
Cross-sectional area intervertebral foramen (CSA-IVF) (mm^2^)	52.35 ± 14.23	84.87 ± 19.34	<0.05
Cross-sectional area of the facet joint (CSA-FJ) (mm^2^)	216.04 ± 28.23	192.64 ± 21.34	<0.05
VAS	8.36 ± 0.65	0.69 ± 0.45	<0.05
ODI	79.56 ± 23.56	10.74 ± 5.67	<0.05

## Discussion

Symptomatic lumbar lateral recess and same-level foraminal stenosis is a lesion that leads to significant disability from both traversing and exiting nerve root dysfunction (14, 15). Decompression with interbody fusion surgery is considered the standard gold treatment for these lesions. However, unfavorable postoperative complications, such as pseudoarthrosis, instrumental failure, and adjacent segment disease, have been reported (16).

Various minimally invasive nonfusion techniques have been developed to solve these problems (7). UBE surgery has significant advantages, such as a good operative view, easy surgical manipulation, reduced blood loss, and decreased postoperative back pain. It has been considered a minimally invasive technique with favorable clinical outcomes and high patient satisfaction (17). For nonfusion endoscopic spinal surgery, the preservation of facet joints on the pathological side is the most crucial consideration (18, 19). Despite UBE surgery leading to less iatrogenic injury due to its flexible manipulation and good visualization, there are still challenges to overcoming the violation of the facet joints in ipsilateral approach surgery (20). As the visualization is limited to the vertical trajectory in the ipsilateral approach, partial resection of the facet joint may be necessary to approach the lateral recess and the foramen. It has been reported that the violation of the medial facet joint is inevitable for adequate exposure to the surgical field in the ipsilateral approach, especially in conditions such as facet hypertrophy combined with foramina stenosis (21).

A contralateral inclinatory approach has been attempted by some authors to overcome the iatrogenic facet violation in the ipsilateral approach (22, 23). Chang et al. (24) reported that the contralateral inclinatory approach can be an effective alternative surgical approach in managing cervical spondylotic radiculopathy in microscopic decompression surgery using a tubular retractor. Kwan-Su Song et al. (25) first introduced contralateral inclinatory cervical foraminotomy by applying the UBE surgery technique to treat cervical radiculopathy pathologies. This approach allowed enough foraminal decompression with less facetectomy without violating the facet capsule compared with conventional ipsilateral UBE surgery, which needed more facetectomy for sufficient foraminal decompression. De Antoni et al. (26) first described the contralateral approach to biportal surgery using arthroscopy with a patient in the lateral position in 1996. However, to our knowledge, there is no description of the merits of contralateral inclinatory approach decompression *via* UBE surgery at the lumbar level with patients in the prone position. In this study, the CIA-UBE technique was applied to acquire a wider operative view of the surgical region, and its results have been reported with successful radiological and clinical outcomes.

In our series, CIA-UBE achieved good clinical and radiological outcomes. All patients had improved leg pain, VAS and ODI values were satisfied with less postoperative leg pain, operative scarring was minimal, and hospital stay was short. Radiological results in this study showed significant enlargement of the lateral recess and foraminal area in all 10 cases and successfully removed protruded discs without compromising the stability of the lumbar spine in six cases. This indicates that CIA-UBE may be a useful technology for foraminal and lateral recess stenosis with facet joint preservation at the lumbar level.

In our described CIA-UBE approach, the surgeon stands on the contralateral side of the lesion, whereas two portals are created at the lesion side over the spinous process. The inclinatory trajectory angle is usually 30–40°, which is between an ipsilateral approach and a contralateral sublaminar approach. An appropriate angle visualization of the surgical field can enable optimal decompression of the lateral recess and same-level foraminal region, which is a significant factor in such successful clinical results in the current cases. Compared to the vertical ipsilateral approach, CIA-UBE enables more incline and a longer trajectory, and the spinal inner space for surgical intervention to the lateral recess and same-level foramen will be proportionally increased. During decompression, endoscopy and the instruments can direct laterally toward the lateral recess and same-level foraminal region, and the plane between the nerve roots and the pathological regions can be visualized from an overhead direction using a 30° endoscope. Compared to the contralateral sublaminar approach, which also can treat the combined lumbar lateral recess and foraminal lesions (13), CIA-UBE provides a more direct and shorter trajectory, which can reduce bone-cutting work and intracanal manipulation.

In addition to adequacy decompression, another important purpose of adopting the CIA-UBE approach is the minimization of violations of the facet joint. During ipsilateral approach decompression, for the vertical trajectory, more of the outer superficial bone needs to be resected before the inner bone can be undercut to expose the lateral recess and foraminal. However, during CIA-UBE decompression, the facet joint could be more effectively preserved by undercutting the facet joint and saving the dorsal portion of the facet capsule in the inclinatory operative trajectory. In our radiological results, the reduction rate of the facet joint plane was calculated at about 10.83%, which was lower than that of the early reported reduction rate of the facet joint after the ipsilateral approach (18).

There are some technical points to contralateral keyhole endoscopic surgery, listed as follows:

1. There are certain limitations associated with CIA-UBE surgery. Various conditions can restrict access when approaching from the pathological side with the surgeon standing on the contralateral side, for example, spinous process deviation toward the pathological side, spinous process hypertropy, and central or extra-foraminal disc herniation. Furthermore, severe degenerative scoliosis with facet arthropathy and the narrow lamina in the upper segments may also block the approach. Therefore, the choice depends on each patient's spinal anatomy and pathology.
2. Another demerit of CIA-UBE is a technical difficulty with a steep learning curve (27). In the process of laminotomy, the initial operative field is relatively narrow, and sometimes, a steep operative angle is needed. In addition, extensive drilling of the facet joint is more possible than the ipsilateral approach for the inclinatory operative trajectory if surgeons are unfamiliar with the anatomic landmark. Thus, surgeons should try this approach after they are familiar with the ipsilateral approach.
3. The skin incisions are suggested to be made obliquely along the multifidus muscle in the vicinity of the spinous process, being 5 mm lateral to the spinous process locking at the spinolaminar junction. If a skin incision is made other than locking at the spinolaminar junction, it is easy to drill out on the contralateral side of the laminar along the inclinatory trajectory, leading to violations of the facet joint and joint capsule.
4. Because the operative field of the primary region is relatively narrow *via* CIA-UBE, to obtain a wider vision, 30° endoscopy was recommended. In addition, angled chisel and bent drills were useful surgical tools to remove the medial part of the lateral recess and the tip of the superior articular process. Therefore, for easy handling of these angled endoscopic instruments, the laminectomy should be made wide enough at the base of the spinous process.

There are several limitations to this study. First, this study is a retrospective study of case series involving a small sample size and having a short follow-up period, which prevented the detection of complications such as the development of segmental instability and recurred disc herniation. Second, although we demonstrated better lateral recess and same-level foraminal stenosis decompression in our cases, most cases (8 of 10) involved a left-sided stand approach (lesions on the right side), which our right-handed surgeon found easier to operate. During the left-sided stand approach, the endoscope can show a more broad and detailed view of the foraminal space than the right-sided stand approach (lesions on the left side) and the instruments can access the foraminal area conveniently and efficiently. The statistics from a right-sided approach were lacking, and this is another limitation of our study. Third, measurement of the reduction rate may be inaccuracte in reflecting facet joint violation with bias. A further follow-up evaluation with a large number of patients would be necessary to prove the efficacy of CIA-UBE in the long term.

## Conclusion

CIA-UBE can provide direct access to the lateral recess and same-level foramen with one window at the lumbar level, avoiding another incision. This approach may also minimize the iatrogenic damages to the facet joint by undercutting the bony structure with an inclinatory approach angle and is worthy of further application.

## Data Availability

The raw data supporting the conclusions of this article will be made available by the authors, without undue reservation.
